# Motion Periods of Planet Gear Fault Meshing Behavior

**DOI:** 10.3390/s18113802

**Published:** 2018-11-06

**Authors:** Mian Zhang, Kesheng Wang, Yaxin Li

**Affiliations:** School of Mechanical and Electrical Engineering, University of Electronic Science and Technology of China, Chengdu 611731, China; zoommian@foxmail.com (M.Z.); liyaxin1205@163.com (Y.L.)

**Keywords:** planetary gearbox, planet gear fault, motion period, fault meshing position, minimal required data length

## Abstract

Vibration sensors are, generally, fixed on the housing of planetary gearboxes for vibration monitoring. When a local fault occurred on the tooth of a planet gear, along with the system operating, the faulty tooth will mesh with the ring gear or sun gear at different positions referring to the fixed sensor. With consideration of the attenuation effect, the amplitudes of the fault-induced vibrations will be time-varying due to the time-varying transfer paths. These variations in signals are valuable information to identify the fault existence as well as the severity and types. However, the fault-meshing positions are time-varying and elusive due to the complicated kinematics or the compound motion behaviors of the internal rotating components. It is tough to accurately determine every fault meshing position though acquiring information from multi-sensors. However, there should exist some specific patterns of the fault meshing positions referring to the single sensor. To thoroughly investigate these motion patterns make effective fault diagnosis feasible merely by a single sensor. Unfortunately, so far few pieces of literature explicitly demonstrate these motion patterns in this regard. This article proposes a method to derive the motion periods of the fault-meshing positions with a faulty planet gear tooth, in which two conditions are considered: 1. The fault-meshing position initially occurs at the ring gear; 2. The fault-meshing position initially occurs at the sun gear. For each scenario, we derive the mathematical expression of the motion period in terms of rotational angles. These motion periods are, in essence, based on the teeth number of gears of a given planetary gearbox. Finally, the application of these motion periods for fault diagnosis is explored with experimental studies. The minimal required data length of a single sensor for effective fault diagnosis is revealed based on the motion periods.

## 1. Introduction

Assembled with a sun gear, several planet gears and a ring gear, planetary gearboxes have brought superior features such as large transmission ratio, high torque to weight ratios and coaxial shafting [1,2,3] in transmission train [4,5,6,7,8]. Commonly, planet gears not only rotate by themselves but also revolve around the sun gear. This typical arrangement, to a great extent, provides load-sharing protection for each planet gear. However, along with the operation of the planetary gearbox, several planet-sun gear pairs and planet-ring gear pairs are meshing simultaneously. Under this circumstance, compound motion behaviors of planet gears give rise to complexity and challenges in fault detection of planetary gearboxes.

The fault detection approach can be mainly divided into the model-based method, signal based method, and data-driven method [9,10]. Vibration monitoring is a popular and effective way to support the above methods [11,12,13,14,15]. Generally, vibration data is captured from an external sensor mounted on the housing to monitor the health conditions of gears. When a local fault occurred on a planet gear, regarding a fixed sensor, the fault-meshing position is time-varying which lead to the time-varying propagating distance from the fault-meshing position to the sensor [16]. With consideration of the attenuation effect, the amplitudes of the fault-induced vibration will contribute stronger when the fault-meshing position reaches closer to the sensor and will be weaker vice versa. These time-varying amplitudes of fault-induced vibrations, which will cause significant changes in signals, are direct fault-related features to identify the fault existence as well as the severity and types. To sufficiently but without excessively taking advantage of these fault information, special attention to the rule of the kinematics of fault-meshing positions needs to be attended so that the entire fault information induced by every possible fault-meshing position can be held.

The vibration response of gearboxes has a strong connection with the meshing behavior of the gears. Concerning a fixed sensor, the faulty tooth on a gear in fixed-shaft gearboxes always meshing at the same position. Therefore, the entire vibrations induced by a faulty gear in fixed-shaft gearbox can be obtained merely within one complete rotation of the faulty gear [17]. For a faulty gear in planetary gearboxes, however, employing rotation cycles of the faulty gear to determine the same fault-meshing position are much more difficult due to the compound motion of internal gears. If a faulty tooth on a planet gear is in meshing at an initial position, after one rotation of the planet carrier, the planet gear returns to its initial position. However, different tooth (not the faulty tooth) of the considered planet gear might be in meshing at the original position [18,19,20,21]. In such a scenario, compound motion behaviors of the rotating components inside planetary gearboxes need to be analyzed in determining a complete pattern of the faulty gear meshing.

P. Samuel et al. [22] and G.D’Elia et al. [23] proposed a minimum required number of rotations for an analyzed tooth on an interesting gear (sun gear or planet gear) that returns to its initial meshing position. However, the required numbers of rotations were computed with the reference of the planet carrier. In other words, it is not applicable to the real measured data from a fixed sensor directly. Additionally, these numbers of rotations lack rigorous mathematical derivations. Afterward, Wang et al. [24] took the fixed sensor as the reference and derived the minimal required number of rotations of a faulty sun gear returning to its initial fault-meshing position. It is a complete period that contains all possible fault-meshing positions refer to the fixed sensor. However, their derivation was solely based upon the teeth number of a given planetary gearbox. Generalized kinematic rule or expressions for the fault-meshing positions of sun gear was not summarized. Song [25] also took the fixed sensor as the reference, who try to derive the motion periods for the faulty tooth of the sun gear or the planet gear returning to its initial fault-meshing positions of a given planetary gearbox. However, the derived periods must base on the prior knowledge of total fault-meshing times when the faulty tooth return to its initial fault-meshing position. This will induce complexity in determining those periods. Moreover, Wang et al. [26,27] investigated the motion behavior of the sun gear faulty tooth. With respect to the fixed sensor, they provided an expression of a motion period to reveal the fault-meshing behaviors. Jong et al. [28,29] proposed the representations of the numbers of carrier rotations to describe a specific ring-planet gear tooth which reset to its initial meshing position. Whereas, analytical procedures of how to get the numbers of rotations in the above articles are missed.

From the above analysis, it is imperative to propose a method which can explicitly interpret the motion behaviors of the fault meshing positions for planetary gearboxes. Thoroughly understanding of these fault meshing behaviors make effective fault diagnosis even with a single sensor come true. In this article, concerning one fixed sensor, the motion behaviors of a local fault on a planet gear are thoroughly analyzed. Due to the unique meshing behaviors of the planet gear, two different conditions of fault-meshing are considered:

1. The faulty tooth initially meshing with the ring gear;
2. The faulty tooth initially meshing with the sun gear.

We propose the generalized expressions of the motion periods including the above two conditions in terms of rotational angles, respectively. Finally, applications of the motion periods for fault diagnosis will be explored through experimental studies. Besides, the derived expressions of the periods will be validated in a geometrical view.

Remaining parts are arranged as follows: Section 2 introduces the transmission effects and derives the motion periods of the planet gear fault-meshing positions. Section 3 applies the experimental studies. Section 4 concludes the whole paper. A vivid geometrical analysis is attached in Appendix A.

## 2. Motion Periods of the Planet Gear Fault-Meshing Position

**Figure 1:**
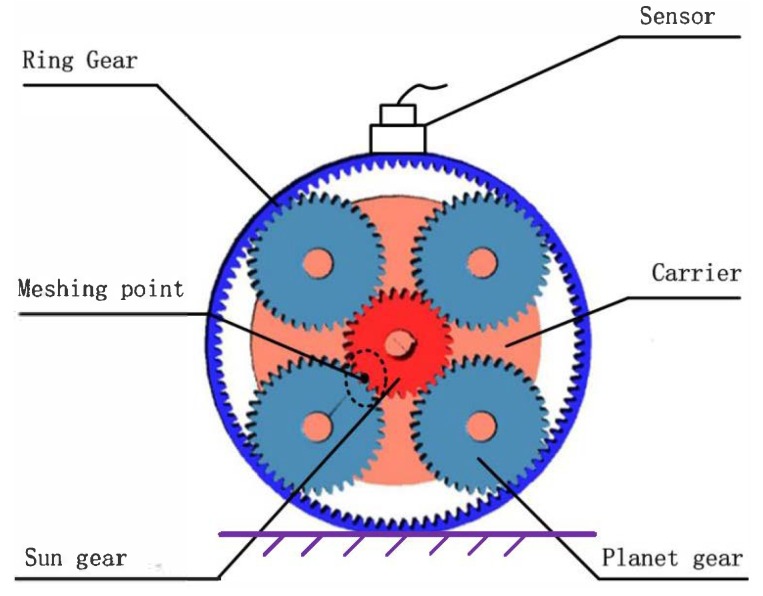
The schematic of planetary gear system.

A typical planetary gearbox will be analyzed in this paper. As is shown in Figure 1, the ring gear is fixed, the sun gear is connected with the shaft as the power input, and the planet carrier is the output. Besides, planet gears are equally assembled, and the vibration sensor is fixed on the housing of the system.

Before investigating the motion period of the fault-meshing positions, effects of the transmission paths will be discussed first so that the motivation of our work can be understood.

### 2.1. Influences of the Fault-Meshing Behavior

**Figure 2:**
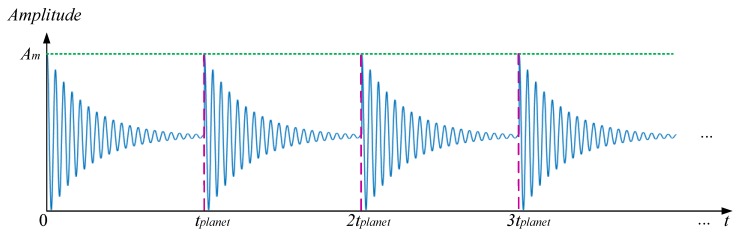
Schematic of the fault induced vibrations, $x_{planet}\left(t\right)$.

When a local fault occurred on a planet gear tooth, a fault induced meshing impact or impulse will be generated and then exhibit in an oscillation form. With consideration of the attenuation effects [30], the fault induced vibration can be represented as follow:

(1) $$x_{planet}\left(t\right)=A_{m}e^{-\xi w_{n}\tau }cos\left(w_{d}t+\varphi \right)$$

where $x_{planet}\left(t\right)$ means the fault induced vibration with the damping effect, $A_{m}$ means the amplitude of fault impulse, ξ means the damping ratio, $w_{n}$ means the nature frequency of the system, $w_{d}=\sqrt{1-\xi^{2}}w_{n}$ means the damped nature frequency, φ means the initial phase and τ means the time series with period of $t_{planet}$ namely,

(2) $$\tau =\tau +t_{planet}$$

where $t_{planet}$ means the interval time between two times of fault meshing. It should be noted that, as the system operating, a series of fault impulses will produce which can be represented as in Figure 2.

**Figure 3:**
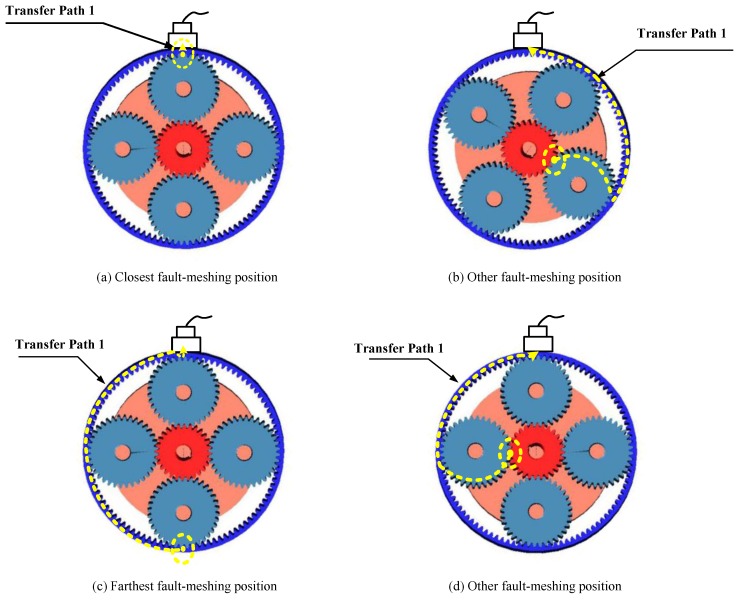
Possible fault-meshing position and the corresponding propagating distance of transfer path1.

Based on [16,24,31], the fault induced vibration will transmit through three different transfer paths to the sensor. Among the three transfer paths, transfer path 2 and 3 do not change along with time [16,31]. The attenuation level of the fault-induced vibration can be deemed identically. The remaining transfer path 1, which depends on the fault meshing-position, need to be concentrated. Once the fault-meshing point moves closest to the sensor, the amplitude of the fault-induced vibration will be stronger due to the least attenuation level of the shortest propagating distances and will be weaker vice versa. We show some possible fault-meshing positions (solid dot) and propagating distances through transfer path 1 (dashed line) to the fixed sensor in Figure 3.

**Figure 4:**
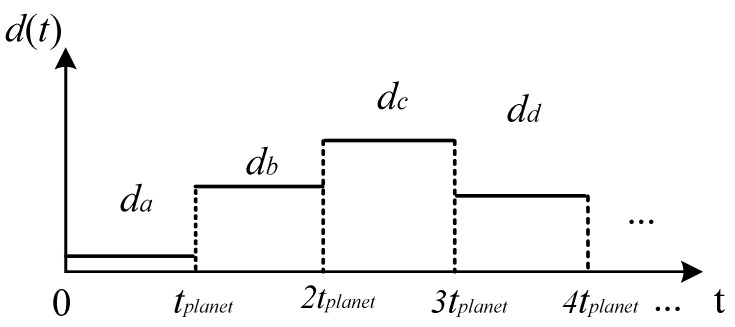
Possible distances between the fault meshing positions and the sensor in Figure 3.

Figure 3a,c exhibits the closest and farthest fault meshing position to the sensor. The captured amplitudes of fault induced vibration will reach maximum and minimum, respectively. Additionally, Figure 3b,d exhibits two possible fault meshing positions neither closet nor farthest to the sensor. Therefore, these two amplitudes of fault induced vibration should fall between the above maximum and minimum values. Suppose using d(t) to represent the distance between the fault-meshing positions, the propagating distances of the fault meshing positions to the sensor in Figure 3 through transfer path 1 can be depicted in Figure 4.

The fault-induced vibration will transmit through d(t) to the sensor so that the sensor captured fault amplitude, $A_{sen}\left(t\right)$, can be represented as:

(3) $$A_{sen}\left(t\right)=A_{m}e^{-\xi w_{n}\frac{d(t)}{v}}$$

where *v* is the propagating speed of the fault induced vibration, $\frac{d(t)}{v}$ means the attenuate time duration from the fault meshing position to the sensor. Notice that for a determined planetary gear system, ξ and $w_{n}$ in Equation (3) should be constant. Besides, the speed of a determined wave, *v* should also be a constant. In such a scenario, the captured fault amplitudes only depend on the propagating distance d(t).

Although it is difficult to accurately determine every fault meshing position so that to determine the time-varying propagating distances, there should exist specific motion patterns of the meshing positions relative to the sensor which satisfy the following criteria:

(4) $$A_{sen}\left(t\right)=A_{sen}\left(t+T_{motion}\right)$$

where $T_{motion}$ represents the motion period of fault meshing positions. Meanwhile, $T_{motion}$ should be the period of d(t). In order to make use of these fault amplitudes for the fault diagnosis of planet gear fault, $T_{motion}$ should be derived as the prior knowledge.

### 2.2. Motion Period of Planet Gear Fault-Meshing Behavior

In this section, the motion behaviors of a single faulty tooth of a planet gear will be discussed. Intuitively, concerning a fixed-sensor, the motion behavior of the planet gear fault-meshing positions should follow some rules. The trouble is that the planet gear faulty tooth may sometimes mesh with the ring gear or sometimes mesh with the sun gear. In this regard, these two conditions will be discussed in the following, respectively.

#### 2.2.1. Initial Fault-Meshing Position at Ring Gear

**Figure 5:**
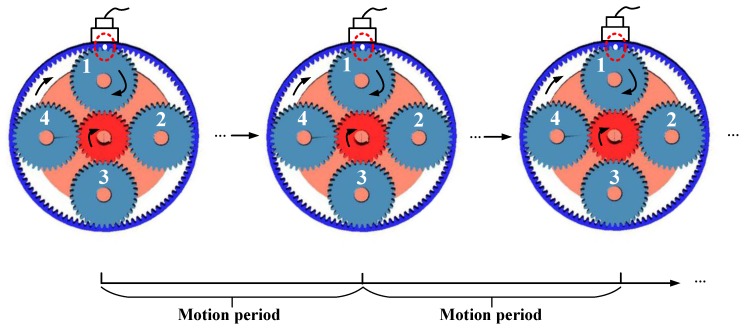
Schematic of the motion period of the initial fault-meshing position at the ring gear.

Assume an initial fault-meshing position that the faulty tooth of a specific planet gear is meshing with the ring gear locating closest in line with the sensor. When the planet gear faulty tooth is again meshing with the ring gear at the same position, a period is completed. Figure 5 illustrates the schematic of this period.

In Figure 5, the solid dot represents the faulty tooth of planet gear 1 is meshing with the ring gear. Rotational directions of the sun gear, the analyzed faulty planet gear, and carrier are also annotated. During one rotation cycle of planet gear 1, the faulty tooth will mesh with the sun gear or the ring gear at different positions. Finally, after several rotations of the planet gear 1 or several rotations of the carrier, a period will complete and then the next period will restart. Inspired by [26], this period is, in essence, similar to the tidal period of the sun gear fault-meshing position. The tidal period of planet gear fault-meshing position should be the analyzed planet gear and carrier both rotating minimal integer cycles, simultaneously. In the following, we will derive this period in terms of rotation angles.

**Figure 6:**
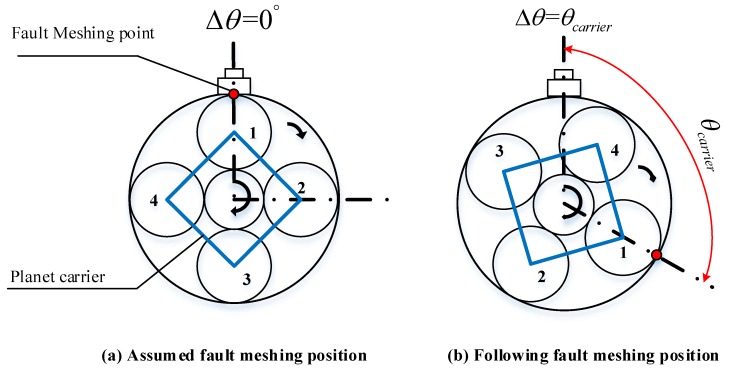
Schematic of the rotation angle between each two times fault-meshing of planet gear 1.

Here, we only consider the fault-meshing position is occurring on the ring gear. In such a scenario, the fault-meshing times only counted once during one complete rotation of the planet gear. Between every two times of the planet gear faulty tooth meshing with the ring gear, we use $\theta_{carrier}$ to represent the rotation angle of the carrier and Δθ to describe the smallest angle between the fault-meshing point to the sensor, both are shown in Figure 6.

In Figure 6, the gears are simplified as circles so that the rotation angles can be conveniently demonstrated. Referring to the fixed sensor, from one fault-meshing point to the next, the rotation angle of planet gear 1, $\theta_{planet}$, must be 2π radians. The time duration between each fault-meshing, $t_{planet}$, can be therefore determined by the rotation angle between two times of fault-meshing divides the angular speed of the planet gear, as is given in Equation (5):

(5) $$\begin{array}{c} t_{planet}=\frac{1}{f_{planet}} \end{array}$$

where $f_{planet}$ means the rotational frequency of the planet gear measured in Hz. The rotation angle of planet carrier between every two times of fault-meshing of planet gear 1, $\theta_{carrier}$, can be determined in the equation below:

(6) $$\begin{array}{cc} \theta_{carrier} & =\omega_{carrier}t_{planet}\\ & =\frac{2\pi f_{carrier}}{f_{planet}} \end{array}$$

where $\theta_{carrier}$ is measured in radians; $\omega_{carrier}$ denotes the angular speed of the carrier measured in radians per second and $f_{carrier}$ denotes the rotational frequency of carrier measured in Hz. Along with the operation of planet gear 1 and the carrier, the faulty tooth meshing with ring gear will occur 1st, 2nd ... etc. times. Eventually, at $m_{ring}$th fault meshing ($m_{ring}$ is an integer), the faulty tooth of planet gear 1 will first return to the initial meshing position as shown in Figure 6a. If we use $\Phi_{planet-ring}$ to represent the total rotation radians of planet gear 1, and $\Phi_{carrier-ring}$ to represent the total rotation radians of the carrier, both total radians are at the $m_{ring}$th fault-meshing:

(7) $$\begin{array}{cc} \Phi_{planet-ring} & =\theta_{planet}m_{ring}\\ & =2\pi m_{ring}\;\; \end{array}$$

and

(8) $$\begin{array}{cc} \Phi_{carrier-ring} & =\theta_{carrier}m_{ring}\\ & =2\pi \frac{f_{carrier}}{f_{planet}}m_{ring} \end{array}$$

where $\Phi_{planet-ring}$ and $\Phi_{carrier-ring}$ are both measured in radians.

According to the criteria of the tidal period of the planet gear fault-meshing positions, the planet gear 1 and the carrier must rotate complete cycles, simultaneously. In other words, this means $\Phi_{planet-ring}$ and $\Phi_{carrier-ring}$ must be an integer number of 2π, simultaneously. Equation (7) tells that for any integer value of $m_{ring}$, $\Phi_{planet-ring}$ is integer number of 2π. However, $\Phi_{carrier-ring}$ from Equation (8), which containing a ratio between $f_{carrier}$ and $f_{planet}$, bring difficulty in determining the integer cycles. According to [22], $f_{carrier}$ and $f_{planet}$ can be established by the gear-meshing frequency of planetary gearboxes, as is shown in the following equation:

(9) $$\frac{f_{carrier}}{f_{planet}}=\frac{Z_{planet}}{Z_{ring}-Z_{planet}}$$

where $Z_{planet}$ and $Z_{ring}$ represent the teeth number of the planet gear and the ring gear, respectively. We substitute Equation (9) into Equations (6) and (8) and then get the expressions of $\theta_{carrier}$ and $\Phi_{carrier-ring}$:

(10) $$\theta_{carrier}=\frac{2\pi Z_{planet}}{Z_{ring}-Z_{planet}}$$

and

(11) $$\Phi_{carrier-ring}=\frac{2\pi Z_{planet}}{Z_{ring}-Z_{planet}}m_{ring}$$

Equations (10) and (11) reveal that between two times of fault-meshing the carrier rotational angle, and total rotational angle of the carrier to be a tidal period, intrinsically depends on the teeth number of planet gear and ring gear. These teeth numbers are necessarily integer number.

Notice that both $\Phi_{planet-ring}$ and $\Phi_{carrier-ring}$ contains an integer term $m_{ring}$, it is reasonable to take a ratio between $\Phi_{planet-ring}$ and $\Phi_{carrier-ring}$ to cancel the common divisor:

(12) $$\frac{\Phi_{planet-ring}}{\Phi_{carrier-ring}}=\frac{n_{planet-ring}}{n_{carrier-ring}}=\frac{Z_{ring}-Z_{planet}}{Z_{planet}}$$

where $n_{planet-ring}$ and $n_{carrier-ring}$ represent the rotational cycles of the faulty planet gear and carrier to be a tidal period (Φ=2πn). Suppose a greatest common divisor of the numerator and denominator exist in Equation (12), namely

(13) $$X_{1}=GCD\left\{Z_{ring}-Z_{planet},Z_{planet}\right\}$$

where GCD{} means the greatest common divisor of the whole terms in the bracket.

Equation (13) tells that, when the planet gear rotates $\frac{Z_{ring}-Z_{planet}}{X1}$ cycles and simultaneously carrier rotates $\frac{Z_{planet}}{X1}$ cycles, a tidal period is completed. Identically, we can also use the least common multiple to represent the rotation cycles in a tidal period:

(14) $$n_{planet-ring}=\frac{LCM\{Z_{ring}-Z_{planet},Z_{planet}\}}{Z_{planet}}$$

and

(15) $$n_{carrier-ring}=\frac{LCM\{Z_{ring}-Z_{planet},Z_{planet}\}}{Z_{ring}-Z_{planet}}$$

The total fault meshing times in a tidal period can be therefore determined based on Equation (14) or Equation (15):

(16) $$m_{ring}=\frac{LCM\{Z_{ring}-Z_{planet},Z_{planet}\}}{Z_{planet}}$$

In this section, with consideration of the initial fault-meshing position that the faulty tooth of a planet gear meshing with the ring gear, the motion period of the fault-meshing positions is derived in terms of the rotation angle, which intrinsically depends on the teeth number of a planetary gearbox. The time duration of this motion period can be determined by the rotational cycles of carrier or planet gear:

(17) $$t_{tidal-ring}=\frac{n_{carrier-ring}}{f_{carrier}}=\frac{n_{planet-ring}}{f_{planet}}$$

where $t_{tidal-ring}$ represents the motion period of the faulty tooth of a planet gear initially meshing at the ring gear. In such a scenario, the period of the fault amplitudes in Equation (4) should be $T_{motion}=t_{tidal-ring}$. This period should be considered during the signal modeling within faulty planet gear so that some novel fault related sidebands can be revealed. In the next section, the motion period of the faulty tooth initially meshing with the sun gear will be discussed.

#### 2.2.2. Initial Fault-Meshing with Sun Gear

**Figure 7:**
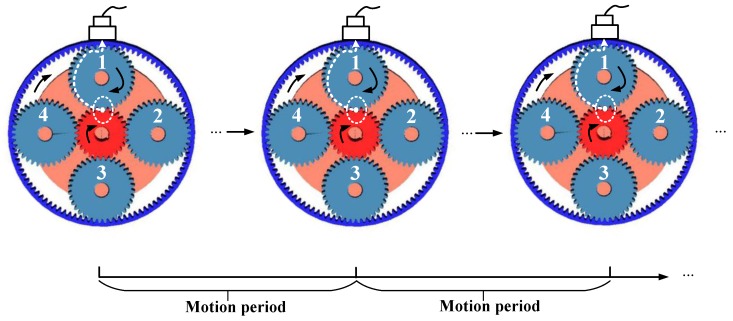
Schematic of the motion period of the initial fault-meshing position at the sun gear.

Now assume the initial fault-meshing position that the faulty tooth of a planet gear meshes with the sun gear locating closest in line to the fixed sensor. When the planet gear faulty tooth again meshing with the sun gear at the same position, a period is completed. We give the schematic of this period in Figure 7.

If each tooth of sun gear is treated identically, the period of the fault-meshing positions equals to $t_{tidal-ring}$ which we have derived in the Section 2.2.1. Because this period only requires the analyzed planet gear and planet carrier rotating complete cycles, simultaneously. In practice, however, imperfections due to the inevitable manufacturing assembly errors or an uneven load or after a period of operation, different sun gear tooth may suffer from different working conditions. The teeth of sun gear should not be treated identically due to the different tooth will result in different vibrations pattern. In this regard, the period of the faulty tooth of a planet gear meshing with a specific tooth of sun gear will be thoroughly discussed.

Inspired by the derived motion period in Section 2.2.1, the motion period of a faulty tooth meshing with a specific sun gear tooth requires the sun gear, the analyzed planet gear, and the carrier rotating integer cycles, simultaneously. We symbolize this typical motion period as $t_{tidal-sun}$ and will also derive this period in terms of rotational angles.

**Figure 8:**
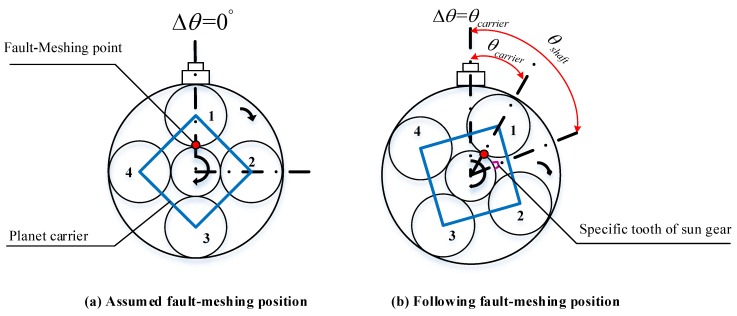
Rotating angle between two times of fault-meshing.

Here, we only focus on the fault-meshing point occurring on the sun gear. Correspondingly, this means the fault-meshing times only be counted once with respect to one complete rotation cycle of the faulty planet gear. Between each two times of the faulty tooth of a planet gear meshing with the sun gear, the rotation angle of the carrier, $\theta_{carrier}$, and the rotation angle of the specific tooth on sun gear, $\theta_{sunrot}$, both are shown in Figure 8.

Between every two times of fault-meshing, the rotation angle of the analyzed planet gear, $\theta_{planet}$, equals to 2π radians; the rotation angle of carrier has been derived in Equation (8); the rotation angle of the specific tooth on the sun gear, $\theta_{sun}$, should equal to the angular speed of sun gear shaft, $\omega_{sunrot}$, multiply by the time duration, $t_{planet}$ in Equation (5), as is given in Equation (18):

(18) $$\theta_{sun}=\omega_{sunrot}t_{planet}=2\pi \frac{f_{sunrot}}{f_{planet}}$$

where $f_{sunrot}$ means the rotational frequency of sun gear shaft which is measured in Hz. The relationship between $f_{sunrot}$ and $f_{carrier}$ can be determined through the meshing frequency [22]:

(19) $$\frac{f_{sunrot}}{f_{carrier}}=\frac{Z_{sun}+Z_{ring}}{Z_{sun}}$$

where $Z_{sun}$ represents the tooth number of the sun gear. According to the machinery handbook [32], the relationship of the teeth number in a planetary gearbox must subject to the following rule:

(20) $$Z_{ring}=Z_{sun}+2Z_{planet}$$

Based on Equations (9), (19) and (20), $\theta_{sun}$ can be rewritten in the following:

(21) $$\begin{array}{c} \theta_{sun}=\frac{4\pi Z_{planet}}{Z_{sun}} \end{array}$$

Equation (21) reveals that $\theta_{sun}$ also only depending on the teeth number of the sun gear and planet gear.

Take Figure 8a as the initial position, assume that after $m_{sun}$ times of fault-meshing, the faulty tooth on planet gear 1 again meshing with the specific tooth of the sun gear. At the same time, the total rotation radians of planet gear 1, the planet carrier and the sun gear can be expressed as follow:

(22) $$\begin{array}{cc} \Phi_{planet-sun} & =\theta_{planet}m_{sun}\\ & =2\pi m_{sun} \end{array}$$

(23) $$\begin{array}{cc} \Phi_{carrier-sun} & =\theta_{carrier}m_{sun}\\ & =\frac{2\pi Z_{planet}}{Z_{ring}-Z_{planet}}m_{sun} \end{array}$$

(24) $$\begin{array}{cc} \Phi_{sun} & =\theta_{sun}m_{sun}\\ & =\frac{4\pi Z_{planet}}{Z_{sun}}m_{sun} \end{array}$$

where $\Phi_{planet-sun}$ means the total rotation radians of the faulty planet gear; $\Phi_{carrier-sun}$ means the total rotation radians and $\Phi_{sun}$ means the total rotation radians of the sun gear, all of them are to be a motion period. Based on the requirement of this motion period, all of the rotational radians must be integer multiple of 2π, simultaneously. A ratio will be taken among Equations (22)–(24) to cancel the common divisor:

(25) $$n_{sun}:n_{planet-sun}:n_{carrier-sun}=\frac{Z_{ring}+Z_{sun}}{Z_{sun}}:\frac{Z_{sun}+Z_{planet}}{Z_{planet}}:1$$

where $n_{sun}$, $n_{planet-sun}$ and $n_{carrier-sun}$ represent the number of rotations of sun gear, faulty planet gear and carrier in a motion period. Actually, determining the minimum integer rotation cycles of the three components is to find the least common denominator of Equation (25) and the number of rotating cycles of the carrier can be determined:

(26) $$\begin{array}{cc} n_{carrier-sun} & =LCD\{\frac{Z_{ring}+Z_{sun}}{Z_{sun}},\frac{Z_{sun}+Z_{planet}}{Z_{planet}},1\}\\ & =\frac{LCM\{Z_{ring}+Z_{sun},Z_{sun}\}}{Z_{ring}+Z_{sun}}\frac{LCM\{Z_{sun}+Z_{planet},Z_{planet}\}}{Z_{sun}+Z_{planet}}\\ & =\frac{LCM\{Z_{ring}+Z_{sun},Z_{sun}\}}{Z_{ring}+Z_{sun}}n_{carrier-ring} \end{array}$$

where LCD means calculating the least common denominator of all the terms in the bracket. Correspondingly, the rotation cycles of the sun gear and planet gear to be a motion period can be determined:

(27) $$n_{sun}=\frac{LCM\{Z_{ring}+Z_{sun},Z_{sun}\}}{Z_{sun}}n_{carrier-ring}$$

(28) $$n_{planet-sun}=\frac{LCM\{Z_{ring}+Z_{sun},Z_{sun}\}}{Z_{ring}+Z_{sun}}n_{plane-ring}$$

The time duration to be a tidal period depend on the number of rotations of the shaft and the input rotational frequency, as is shown:

(29) $$t_{tidal-sun}=\frac{n_{sun}}{f_{sunrot}}=\frac{n_{planet-sun}}{f_{planet}}=\frac{n_{carrier-sun}}{f_{carrier}}$$

where $t_{tidal-sun}$ means the time duration of a motion period in which the initial fault-meshing position at sun gear. In such a scenario, the motion period of fault induced amplitudes in Equation (4) should be $T_{motion}=t_{tidal-ring}$. This period considering the sun gear teeth are different from each other and more complete fault induced information is covered. Therefore, the vibration data measured in this period can be used to diagnostic compound faults, such as the faults occurred on both sun gear and planet gear teeth effectively.

In Section 2.2.1 and Section 2.2.2, two typical motion periods for the faulty tooth of the planet gear are discussed. These periods, which intrinsically based on the teeth number of planetary gearboxes, are unique natures to describe the kinematic motion behaviors of the fault-meshing positions. Since the time duration of $t_{tidal-ring}$ covers entire fault induced vibration by all the possible fault meshing positions, we will discuss the application of this period for fault diagnosis by experimental studies.

## 3. Experimental Study

**Figure 9:**
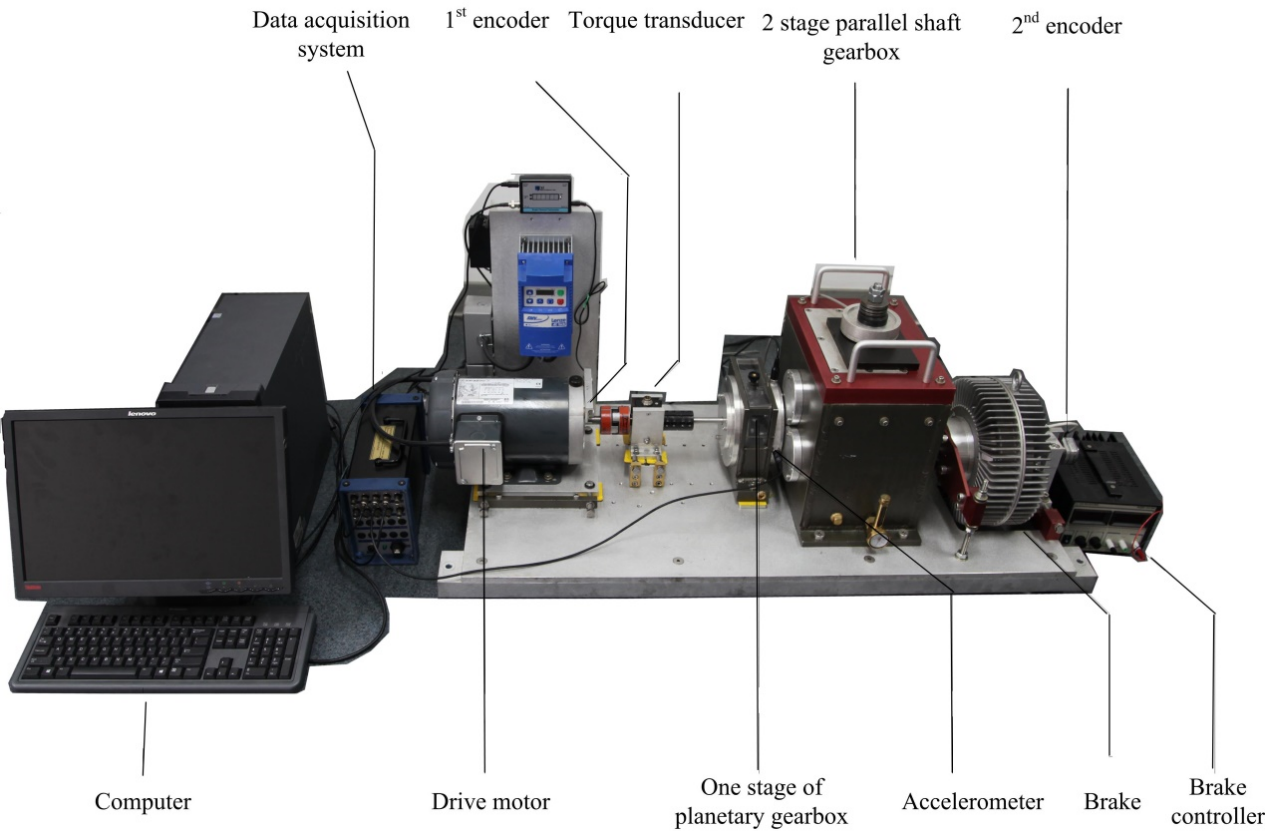
Experimental test rig.

The experimental data are collected from a planetary gearbox test rig at University of Electronic Science and Technology of China (UESTC), Equipment Reliability and Prognostic and Health Management Laboratory (ERPHM). The configuration of the test rig is shown in Figure 9. More detail information can be obtained in [1]. Geometry parameters of the planetary gearbox are listed in Table 1.

**Figure 10:**
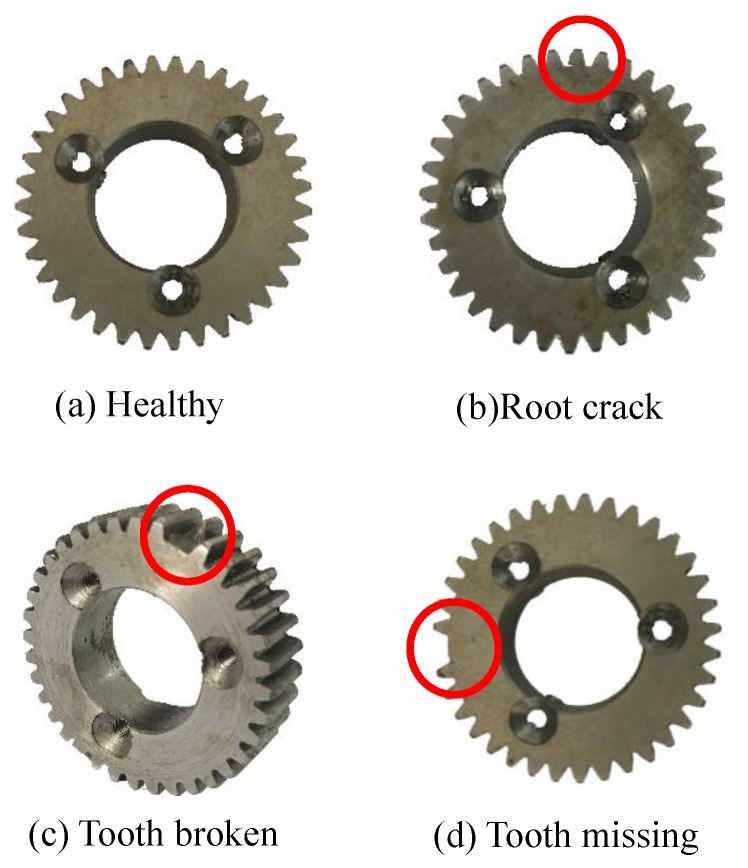
Planet gear health scenarios.

The experiments are operated on the planetary gearbox, using an accelerometer to collect the data. Four planet gear fault scenarios are considered, as is shown in Figure 10.

For each health scenario, 30 groups of data are acquired under the rotational speed of 1800 rpm and 3000 rpm, the data length of 13.8 s, and sampling frequency of 7680 Hz. Since the sun gear is in full healthy state, we may explore the diagnostic ability of the difference data length selected based on $t_{tidal-ring}$. According to Table 1 and Equation (17), $t_{tidal-ring}$ can be determined in Table 2:

Each signal will be sliced into different lengths, namely $\frac{1}{4}t_{tidal}$, $\frac{1}{2}t_{tidal-ring}$, $t_{tidal-ring}$ and $2t_{tidal-ring}$. For each signal pieces, we use two simple indicators, namely root mean square (RMS) and Kurtosis to explore the diagnostic effects of those data lengths.

**Figure 11:**
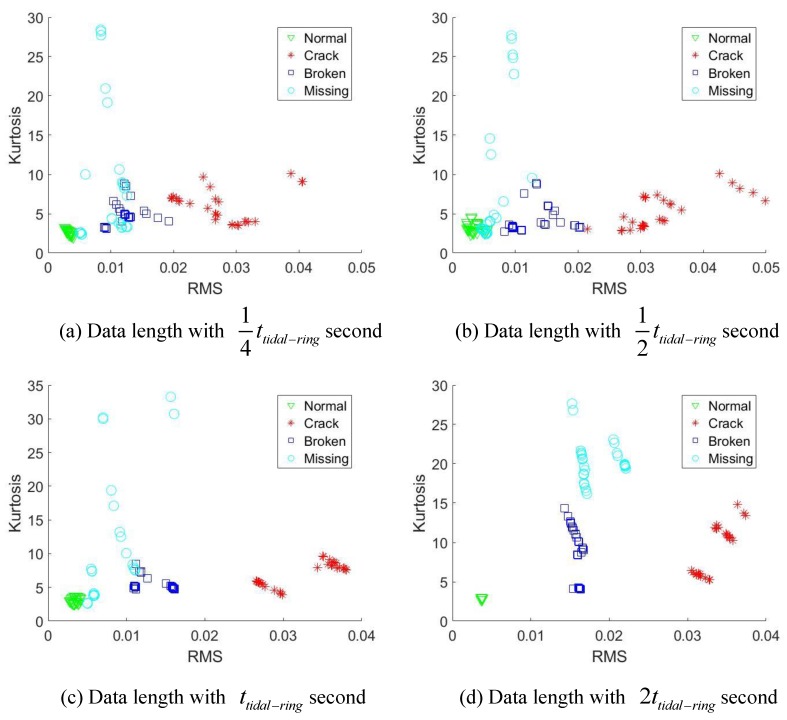
Diagnostic effects under rotational speed of 1800 rpm.

**Figure 12:**
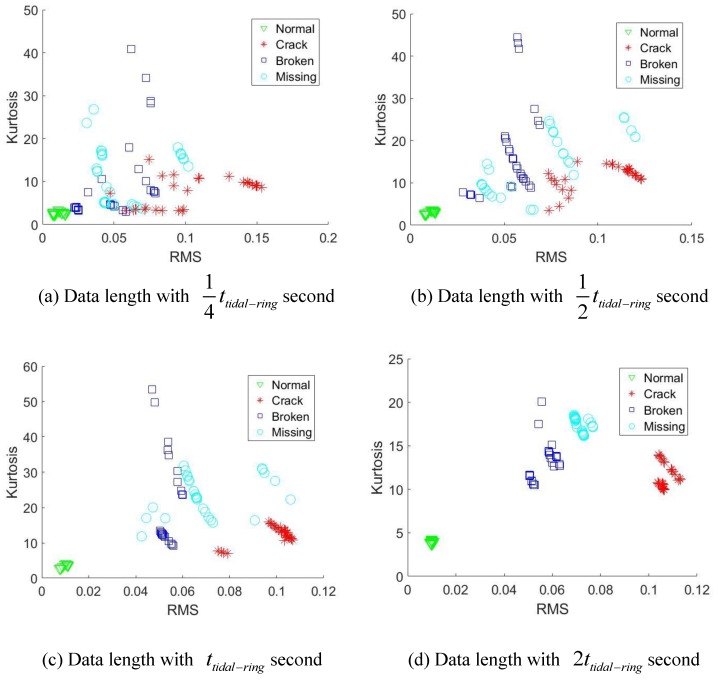
Diagnostic effects under rotational speed of 3000 rpm.

From Figure 11a–c and Figure 12a–c, the data length with $\frac{1}{4}t_{tidal-ring}$, $\frac{1}{2}t_{tidal-ring}$, and $t_{tidal-ring}$ can not separate all the health scenarios clearly. A trend can be discovered that as the data length becomes longer, the better fault separation ability will be obtained due to more fault induced information is covered. Finally, Figure 11d and Figure 12d both reveal that, when the data length achieve 2 times of $t_{tidal-ring}$, the health scenarios are clearly identified. This is because the initial fault meshing positions for different signal segments may be different relative to the sensor. 2 times of $t_{tidal-ring}$ measured data length guarantee that each signal segment covers the fault induced vibration by all the possible fault meshing positions. Consequently, 2 times of $t_{tidal-ring}$ data length can be used as the minimal required analyzed data length for effective fault diagnosis of the planet gear fault.

## 4. Conclusions

In this paper, a method is originally proposed to determine the motion periods of the planet fault meshing positions. Due to the unique initial fault meshing position of a faulty planet gear, two scenarios are considered, namely: 1. Initial fault meshing position occurs at the ring gear; 2. Initial fault meshing position occurs at the sun gear. The motion periods are summarized in terms of rotation cycles, and we again highlight them in the following:

- Condition 1: Fault meshing initially occurred on the ring gear
  (30) $$n_{planet-ring}=\frac{LCM\{Z_{ring}-Z_{planet},Z_{planet}\}}{Z_{planet}}$$
  (31) $$n_{carrier-ring}=\frac{LCM\{Z_{ring}-Z_{planet},Z_{planet}\}}{Z_{ring}-Z_{planet}}$$
  (32) $$t_{tidal-ring}=\frac{n_{planet-ring}}{f_{planet}}=\frac{n_{carrier}}{f_{carrier}}$$
- Condition 2: Fault meshing initially occurred at the sun gear
  (33) $$n_{sun}=\frac{LCM\{Z_{ring}+Z_{sun},Z_{sun}\}}{Z_{sun}}n_{carrier-ring}$$
  (34) $$n_{planet-sun}=\frac{LCM\{Z_{ring}+Z_{sun},Z_{sun}\}}{Z_{ring}+Z_{sun}}n_{planet-ring}$$
  (35) $$n_{carrier-sun}=\frac{LCM\{Z_{ring}+Z_{sun},Z_{sun}\}}{Z_{sun}}n_{carrier-ring}$$
  (36) $$t_{tidal-sun}=\frac{n_{sun}}{f_{sunrot}}=\frac{n_{planet-sun}}{f_{planet}}=\frac{n_{carrier-sun}}{f_{carrier}}$$

These periods, which intrinsically depend on the teeth number of gears, are unique natures to reflect faulty planet gears of planetary gearboxes. With these prior fault motion periods, it is more reasonable to diagnosis faults by a single sensor. Experimental studies demonstrate the minimal required analyzed data length, namely $2t_{tidal-ring}$, achieve effective fault diagnosis of planet gear fault. This criteria can be widely applied for the ring-gear fixed planetary gear system and have great potential for the industrials.

## Figures and Tables

**Table 1:** Parameters of planetary gearbox.

$Z_{\mathrm{sun}}$	$Z_{\mathrm{planet}}$	$Z_{\mathrm{ring}}$	*N*
28	36	100	4

**Table 2:** Time duration of $t_{tidal-ring}$.

Rotational Speed	Time Duration of $t_{\mathrm{tidal}-\mathrm{ring}}$
1800 rpm	$\frac{48}{35}$ s
3000 rpm	$\frac{144}{175}$ s

**Figure A1:**
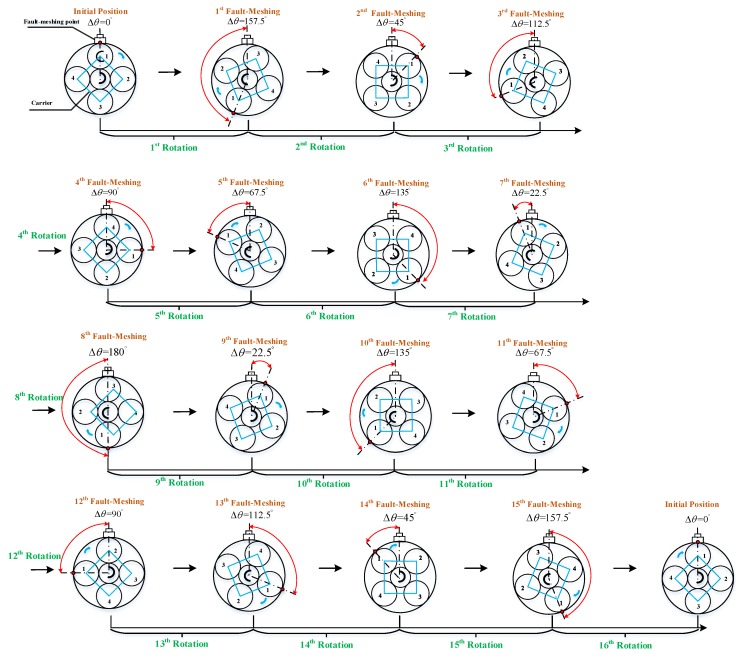
Each fault meshing postion in a motion period.

**Figure A2:**
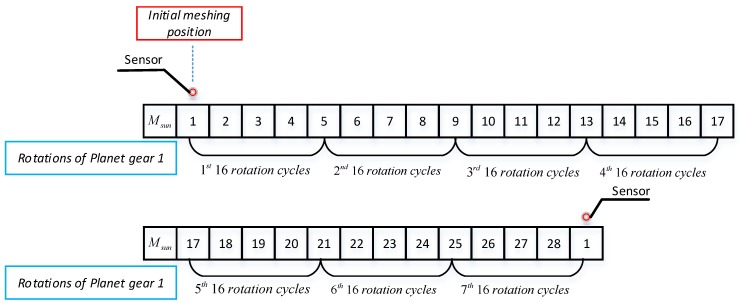
Tooth sequence of the sun gear.

## Abbreviations

The following abbreviations are used in this manuscript:

GCD	Greatest Common Divisor
LCM	Least Common Multiple

## Appendix A. Geometrical validation of the motion periods

The appendix mainly using the geometrical analysis to validate the derived expressions of motion periods with our experimental planetary gearbox. Once a planetary gearbox is determined, some parameters, such as the rotation angles between every two times of fault-meshing could be determined based on Table 1. We list the values of those known parameters, $\theta_{carrier}$, $\theta_{planet}$, $\theta_{sun}$ and values of GCD in Table A1.

**Table A1:** Parameters which can be determined by a planetary gear system.

Known Parameters	Values/Unit	Description
$\theta_{carrier}$	$\frac{9}{16}/cycle$	Rotation angle of carrier between two times of fault-meshing
$\theta_{planet}$	1/cycle	Rotation angle of the faulty planet gear between two times of fault-meshing
$\theta_{sun}$	$\frac{8}{7}$/cycle	Rotation angle of sun gear between two times of fault-meshing
$LCM\{Z_{ring}-Z_{planet},Z_{planet}\}$	576	LCM between $Z_{planet}$ and $\left(Z_{ring}-Z_{planet}\right)$
$LCM\{Z_{ring}+Z_{sun},Z_{sun}\}$	896	LCM between $2(Z_{planet}+Z_{sun})$ and $Z_{sun}$

Additionally, for different initial fault-meshing conditions, some key parameters namely, $m_{ring}$, $n_{planet-ring}$, $n_{carrier-sun}$ as well as $n_{planet-sun}$, are listed in Table A2 and Table A3 for validation.

**Table A2:** Parameters which need to be validated—Initial fault meshing position at ring gear.

Undetermined Parameters	Values/Unit	Description
$m_{ring}$	16/times	Total fault-meshing times of a motion period
$n_{planet-ring}$	16/cycles	Number of rotations of the faulty planet gear in a motion period
$n_{carrier-ring}$	9/cycles	Number of rotations of carrier in a motion period

**Table A3:** Parameters which need to be validated—Initial fault meshing position at sun gear.

Undetermined Parameters	Values/Unit	Description
$m_{sun}$	112/times	Total fault-meshing times of a motion period
$n_{sun}$	288/cycles	Number of rotations of the sun gear in a motion period
$n_{carrier-sun}$	63/cycles	Number of rotations of carrier in a motion period
$n_{planet-sun}$	112/cycles	Number of rotations of faulty planet gear in a motion period

We first validate the correctness of the values in Table A2, a geometrical derivation is demonstrated to track the trace of the fault-meshing point until a period is achieved. Take Figure 6a as the initial position, namely the faulty tooth of sun gear is meshing with ring gear which locating closest to the sensor. Based on the known rotation angles in Table A1, during one rotation cycle of the planet gear 1, the carrier will rotate $\frac{9}{16}$ cycle (202.5${}^{\circ }$). We use Δθ to represent the smallest angle degree between the fault-meshing position and the sensor so that the fault-meshing position can be mathematically determined. Figure A1 gives the each fault-meshing position in a motion period.

Figure A1 reveals that, for the initial fault-meshing position at the ring gear, after planet gear 1 rotates 16 cycles, or fault-meshing occurred 16 times, the faulty tooth of planet gear 1 first return to its initial meshing-position. The result of the geometrical demonstration is identical with the results $n_{planet-ring}$ and $m_{ring}$ which derived by the number of teeth in Table A2.

Now we will validate the correctness of the derived values in Table A3. Since the sun gear has 28 teeth, we symbolize the teeth with the number from 1 to 28. Take Figure 8a as initial fault-meshing position, namely the faulty tooth of planet gear meshing with tooth number ’1’ of the sun gear. When the faulty tooth of planet gear 1 first return to the initial meshing-position, namely the planet gear rotates 16 cycles, the number of rotations of the sun gear, $n_{sun-p}$, can be determined based on Equation (33):

(A1) $$n_{sun-p}=\frac{2Z_{planet}}{Z_{sun}}16=41\;\frac{1}{7}$$

At the same time, the symbolized tooth number of the sun gear, $T_{sun-1}$, which is being meshed with the faulty tooth at the initial position can be determined based on Equation (A1) and $Z_{sun}$:

(A2) $$T_{sun-1}=mod\left(n_{sun-p}Z_{sun},Z_{sun}\right)+1=5$$

where mod(a,b) means the reminder of a/b.

Equation (A2) tells that when the planet gear 1 rotates 16 cycles, the faulty tooth will mesh with the tooth number ’5’ of sun gear at the initial fault position. After 2nd 16 rotation cycles of planet gear 1, the faulty tooth will mesh with the tooth number of $T_{sun-2}$, in which,

(A3) $$T_{sun-2}=mod\left(n_{sun-p}Z_{sun},Z_{sun}\right)+T_{sun-1}=9$$

Equation (A3) reveals that after 2nd 16 rotations cycles or 32 rotation cycles of planet gear, the faulty tooth will mesh with the tooth number ’9’ at the initial position. In the same token, Figure A2 gives the sun gear tooth sequence that each time the faulty tooth of planet gear return to the initial position. Where $M_{sun}$ represents the tooth sequence of the sun gear.

Figure A2 tells that if the initial fault-meshing position that the faulty tooth of planet gear 1 with tooth number ’1’ of sun gear, after 7th 16 rotation cycles or 112 rotation cycles of planet gear 1, the tooth ’1’ of sun gear first return to the initial position. The total fault-meshing, $m_{sun}$, occurred 112 times on the sun gear. The result of geometrical analysis is in full agreement with the $n_{planet-sun}$ in Table A3. The derivation of the tidal period of faulty planet gear is validated.
